# Physical Activity, Sedentary Behaviour and Sleep, and Their Association with BMI in a Sample of Adolescent Females in New Zealand

**DOI:** 10.3390/ijerph17176346

**Published:** 2020-08-31

**Authors:** Lauren S. Tye, Tessa Scott, Jillian J. Haszard, Meredith C. Peddie

**Affiliations:** Department of Human Nutrition, University of Otago, Dunedin 9016, New Zealand; tyela458@student.otago.ac.nz (L.S.T.); tessa.scott@otago.ac.nz (T.S.); jill.haszard@otago.ac.nz (J.J.H.)

**Keywords:** 24-h activity, overweight, obesity, accelerometry, teenager

## Abstract

Despite activity guidelines moving towards a 24-h focus, we have a poor understanding of the 24-h activity patterns of adolescents. Therefore, this study aims to describe the 24-h activity patterns of a sample of adolescent females and investigate the association with body mass index (BMI). Adolescent females aged 15–18 years (*n* = 119) were recruited across 13 schools in 8 locations throughout New Zealand. Actigraph GT3X+ accelerometers were worn 24-h a day for seven days and the output was used to identify time spent in each 24-h component (sleep, sedentary, light-intensity physical activity and moderate-to-vigorous intensity physical activity). In a 24-h period, adolescent females spent approximately half their time sedentary, one third sleeping and the remainder in light-intensity physical activity (15%) and moderate-to-vigorous intensity physical activity (5%). Higher BMI z-scores were associated with 16 min more time spent in light-intensity physical activity. Additionally, those with higher BMI were less likely to meet the sleep and physical activity guidelines for this age group. Compliance with the moderate-to-vigorous intensity physical activity guidelines, sleep guidelines, or both, was low, especially in those classified as overweight or obese. The association between BMI and light activity warrants further investigation.

## 1. Introduction

Physical activity research in children and adolescents has traditionally focused on the effects of participation in moderate-to-vigorous physical activity (MVPA), the behavior that accounts for the smallest time proportion of each day, even in those who participate in the highest levels [1]. Participation in regular physical activity is a well-established preventative measure for several non-communicable diseases such as cardiovascular disease, hypertension, type two diabetes mellitus and some cancers [2]. Recently, there was a shift in focus to acknowledge all the movement behaviors in a 24-h period (sleep, sedentary time and light physical activity (LPA) and MVPA) and how these behaviors either alone, or possibly more appropriately, in combination, can affect health [1,3].

The time components in one 24-h period are co-dependent so that a change in time spent in one movement must influence time spent in another, thus supporting the importance of an integrated movement behavior model [1]. In recognition of the small but growing level of evidence, in 2016 Canada developed 24-h movement guidelines for children and youth [3], which were subsequently adopted by countries including New Zealand [4] and Australia [5]. Accordingly, the guidelines recommend children and youth aged 5–17 years should accumulate at least 60 min of MVPA, attain at least 9–11 h sleep (5–13 years) or 8–10 h sleep (14–17 years) and engage in less than two hours sedentary screen-time, each day. 

In New Zealand, just under 20% of those aged 15–24 are classified as obese [6]. This rate seems comparable to rates reported for the USA and Australia [7,8], although it is slightly higher than the 15% reported for this age group in the UK [9]. Adolescence is a time of growth and development and arguably the most critical period for the prevention of chronic disease as lifestyle behaviors may set healthy patterns for adulthood [10]. Yet, research shows adolescents are displaying high rates of physical inactivity and sedentary time [11,12], poor sleep hygiene [13] and overall poor adherence to the guidelines [11]. A good body of evidence suggests that each of the behaviors that make up a 24-h period (increased sleep, decreased sedentary time and increased physical activity) are associated with decreased odds of obesity and other cardio-metabolic diseases [14]. Research has examined these behaviors in isolation, but there is limited information on the relationship between movement behaviors in a 24-h period and even less evidence for priority groups, such as female adolescents who tend to participate in lower amounts of physical activity than their male peers [15]. Additionally, there appears to be differences in how female adolescents accumulate physical activity across the week [16]; however, to date very few studies have reported 24-h activity patterns individually across days of the week. Therefore, the purpose of this study was to describe the 24-h activity patterns: sleep, sedentary behavior and physical activity, measured in adolescent female participants from the SuNDiAL (The Survey of Nutrition, Dietary Assessment and Lifestyles) project across days of the week; and to assess 24-h behavior compositions in relation to body mass index (BMI) z-score. 

## 2. Materials and Methods

The data used in this study were collected as part of the larger SuNDiAL (The Survey of Nutrition, Dietary Assessment and Lifestyles) project. The study was a nationwide cross-sectional survey of female adolescents in New Zealand (NZ). The main aim of this study was to compare the dietary intakes and habits, nutritional and health status, motivations, attitudes and lifestyles of vegetarian and non-vegetarian adolescent (aged 15–18 years) females in NZ. The sample size required to answer the primary objectives was 300 high school students. A detailed summary of the methods is reported elsewhere [17]. The study was approved by the University of Otago Human Ethics Committee (Health):H19/004. Online informed consent was obtained from all participants and from parents/guardians of those under 16 years. The trial was registered with the Australian New Zealand Clinical Trials Registry: ACTRN12619000290190. 

Initially, high schools in each of the eight predetermined data collection locations across New Zealand were invited to participate by email. These eight locations (Dunedin, Christchurch, Nelson, Wellington, Tauranga, New Plymouth, Whangarei and Central Otago) were chosen based on the living arrangements of the data collectors, who were second-year Masters of Dietetic students from the University of Otago, Dunedin. The recruitment of thirteen schools allowed each pair of data collectors to collect data from one school. Adolescent girls were then recruited from the schools through presentations to classes, year groups or the whole school. Recruitment occurred at two time points, from February to April 2019 and July to September 2019. Adolescents aged 15 to 18 years of age who self-identified as female, could speak and understand English and were not knowingly pregnant, were eligible to participate. 

Most of the data collection was conducted at school during school hours. Prior to the initial assessment appointment, participants provided consent and completed a questionnaire via an online REDCap survey [18]. The questionnaire included basic demographic and health questions, as well as dietary patterns and attitudes and motivations towards food choice [17]. At the initial in-school assessment, weight (measured using one of Medisana PS420; Salter 9037 BK3R; Seca Alpha 770; or Soehnle Style Sense Comfort 400 scales) and height (measured using a Seca 213 or a Wedderburn stadiometer) were taken in duplicate with the participant wearing light clothing, but no footwear, and recorded to the nearest 0.1 kg or cm (respectively). Prior to data collection, an inter-rater reliability study was performed to ensure consistency between data collectors, which showed very good reliability (intra-class correlation coefficients (ICC) of 1.0 for weight and 0.92 for height). BMI was then converted to z-scores using the WHO child growth standards [19], participants were classified as overweight if they had a BMI z-score greater than or equal to one. 

Upon completion of the initial assessment, participants who had consented to wear an accelerometer were fitted with an Actigraph GT3X+ (Actigraph LLC, Pensacola, FL, USA) to assess time spent asleep, in sedentary time or engaged in light or moderate-to-vigorous physical activity. The GT3X+ was initialized to record activity at a sampling rate of 30 Hz without the low-frequency extension. Accelerometers were worn on an elasticated belt over the right hip continuously for 24-h a day for seven consecutive days. Participants were instructed to remove accelerometers during showering, swimming and for high contact sports such as rugby. Over the 7-day wear period, participants were instructed to complete a wear-time and sleep diary. Participants recorded accelerometer removal times and reasons, which were used to identify non-wear time. Activity duration and intensity for activities, such as swimming, that were performed during non-wear time, were also recorded and then manually imputed (manually imputed data accounted for 0.5% of total wear time). Time they got into bed, time they attempted to sleep, estimated time it took to fall asleep (min), as well as time they woke up and time they got out of bed each day were recorded and used to constrain the algorithm used to identify sleep. At the end of the wear period, participants returned their accelerometers and wear-time diary to their school where they were subsequently collected by the data collection team operating in that area.

Days were only considered valid if: 1) wear time during waking hours ≥ 10 h; 2) total wear time or wear time plus imputed physical activity ≥ 20 h; and 3) the day included ≥ 2 h of sleep. A participant’s data were only included in the analysis if there were at least three valid days of data available (as this is the most commonly used requirement [20]). Data were downloaded using Actilife software (Version 6). Data were saved in 15-second epochs, then converted to CSV file and assessed using Stata. Data were collapsed into 1 min epochs and time spent sedentary was classified as less than 150 counts per minute (cpm) using the *y*-axis. Time spent in LPA and MVPA was identified using a threshold of 150 [21] to 1951 cpm and at least 1952, respectively, using the *y*-axis thresholds [22]. Time spent asleep was identified using the Sadeh algorithm [23], which while validated for accelerometer wear on the wrist has been shown to perform well with hip worn accelerometer data, particularly when it is constrained by bedtimes [24]. The algorithm was programmed to only identify sleep between the reported time sleep was attempted and the wake up time reported in the wear time diary. Any physical activity completed when the participant was not wearing an accelerometer was identified as “non-wear time physical activity”. Non-wear time physical activity was added to the amounts of wear time MVPA and LPA depending on the self-reported intensity, to give total MVPA and LPA. The amount and duration of breaks in sedentary time were defined as any occasion when the counts per minute increased above 150 cpm for longer than a minute. A prolonged sedentary bout was defined as an occasion that was ≥30 min of uninterrupted sedentary time. 

All analyses were carried out using Stata (version 16.1 for Mac; StataCorp, College Station, Texas). Mean time spent in sleep (a.m. and p.m.), sedentary time, LPA, MVPA and non-wear time was calculated and expressed as an absolute time and as a percentage of the 24-h day. The average composition of the day is reported using medians (and 25th and 75th percentiles) and the median composition for each day of the week is also presented graphically. All variables are presented as overall medians and individual means for each of the days of the week. To determine the difference in time spent in each component of the day by BMI z-score or by overweight or obese weight category (compared to healthy weight category), mixed effects regression models were used, with the time component as the dependent variable, and BMI z-score or weight status as the independent variable. School clusters were included as a random effect. Regression coefficients and 95% confidence intervals were calculated. Residuals of models were plotted and visually assessed for homogeneity of variance and normality. Proportion of those meeting the sleep and physical activity guidelines were calculated for each BMI category and expressed as a percentage. 

## 3. Results

### 3.1. Participants

**Figure 1:**
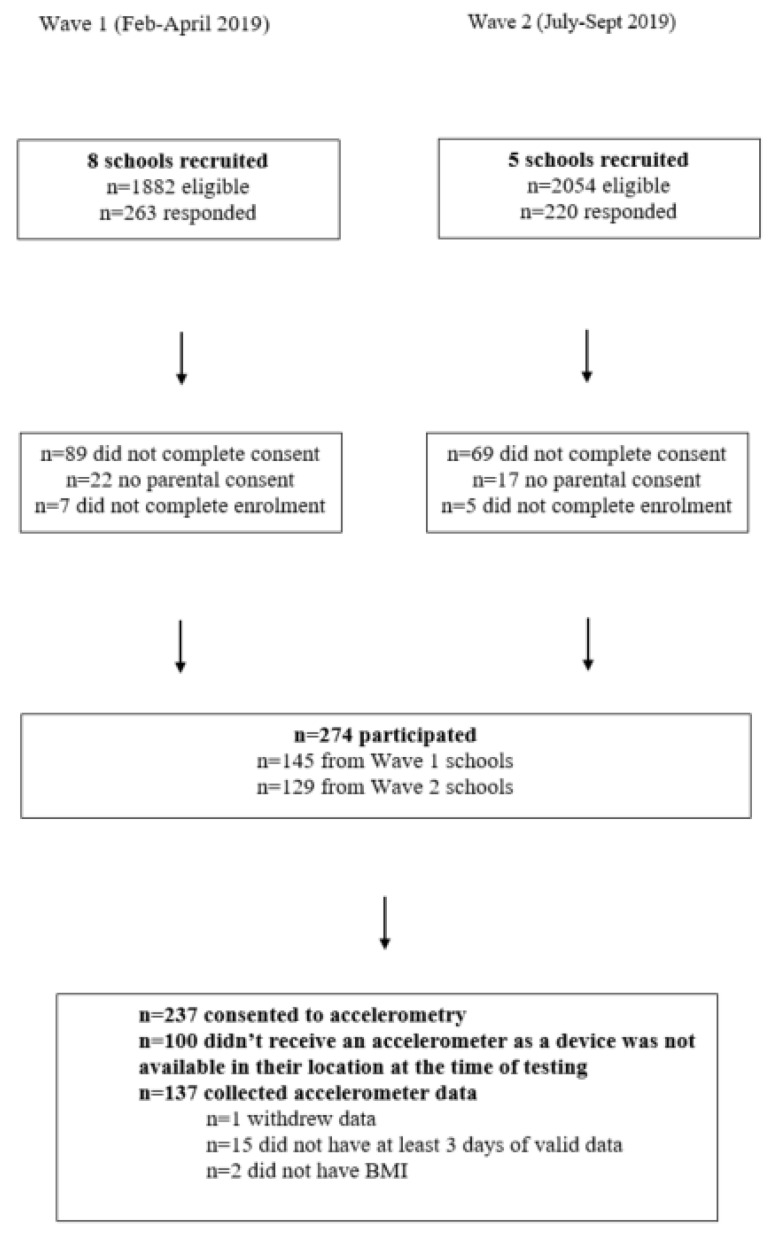
Participant flow.

Of the 274 participants recruited from high schools who completed enrolment and consent, 137 wore accelerometers and 119 provided valid data (Figure 1). The mean (SD) age of participants was 16.8 (0.9) years, 85% of the sample lived in areas of low to moderate deprivation and 66% were categorized as having a healthy BMI [19]. The participants included here (*n* = 119), compared to those who were not (*n* = 151) were not different in terms of age, but were slightly less likely to be of low deprivation (37.0% compared to 43.1%), or obese (7.6% compared to 14.1%). The median number of valid days of accelerometer data was six days. Median (IQR) wear time across valid days was 23.7 (23.4 to 24.0) h.

### 3.2. 24-h Activity Patterns

**Figure 2:**
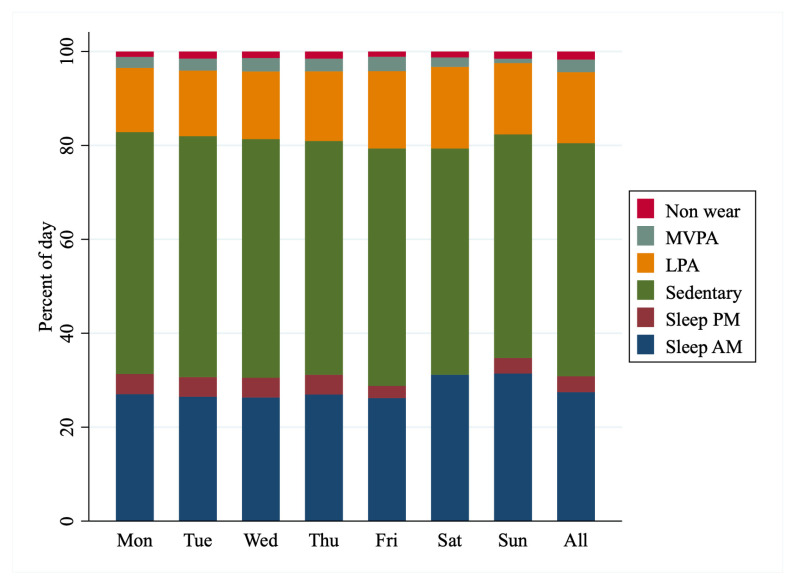
Median time spent sleeping, sedentary and engaging in light physical activity (LPA) or moderate-to-vigorous physical activity (MVPA) across days of the week, as measured by accelerometry in 119 New Zealand adolescent females. Number of valid days for each of the days of the week are as follows: Monday *n* = 94, Tuesday *n* = 90, Wednesday *n* = 68, Thursday *n* = 81, Friday *n* = 90, Saturday *n* = 100, Sunday *n* = 107.

Time spent asleep, in sedentary behavior or engaging in LPA or MVPA are presented in Figure 2. On average, participants spent 30% of their day asleep, 49% sedentary, 15% in light activity and 3% in MVPA. Clear differences in activity accumulation existed according to day of the week, with less MVPA occurring on a Sunday and less total sleep occurring on Friday, while more than half the sample did not record any sleep before midnight on a Saturday night. 

The median (IQR) number of breaks in sedentary time was 62 (54, 68) with the median duration of each break being 13 (12, 15) min. More than half the sample only accumulated one prolonged sitting bout a day, with the median duration of that bout being 60 (51 to 81) min. MVPA tended to accumulate in multiple (median *n* = 15 (12, 20)) very short (3.2 (2.6, 3.8) min) bouts throughout the day.

### 3.3. Associations Between 24-h Components and BMI

The relationship between weight status and 24-h components are presented in Table 1. Overall, there were no meaningful differences in time spent sedentary, asleep or in MVPA by weight status category. However, every 1 BMI z-score higher was associated with 16 min (95% CI: 3,29 min) more light intensity activity and a 156 cpm (95% CI: 10,301) lower intensity of bouts of continuous MVPA.

### 3.4. Adherance to Guidelines

Overall, just 20% (23/119) of the sample were meeting the sleep recommendations for this age group, 23% (27/119) were meeting the MVPA guidelines and only 7% (8/119) participants were meeting both the sleep and physical activity guidelines. There were notable differences between weight status and prevalence of those meeting either the sleep or MVPA guidelines, or both (Table 2).

## 4. Discussion

This is the first study to describe the 24-h activity patterns of a sample of adolescent females aged 15–18 years, in New Zealand. The results indicate that these females spent almost half their time sedentary (49.1%), one third sleeping (30.8%) and the remainder of time engaging in LPA (15%) or MVPA (2.7%). While the amount of time spent sedentary is somewhat alarming, sedentary time is not, for the most part, accumulated in prolonged bouts. Interestingly, MVPA was also accumulated in numerous (median = 15) short (3.2 min) bouts across the day. These results add to the abundance of available evidence indicating adolescent females are not accumulating activity in a manner that is likely to induce health benefits [25]. There are minimal similarly designed studies that measure 24-h activity patterns using accelerometers. Carson et al. [26] found that 6–17-year-olds spent 38% of time sedentary, 40% sleeping and the remainder in LPA (18%) and MVPA (4%). Differences compared to the current study, especially in sedentary time and sleep, are most likely attributable to the inclusion of 6 to 14-years-olds, as movement behaviors often change as a function of age [3]. In adults, Chastin et al. [27] found time was mainly spent sedentary (40%), followed by sleep (28%), LPA (29%) and MVPA (3%). Differences in sleep and MVPA between the current study and the Carson et al. [26] and Chastin et al. [27] studies are probably what would be expected in this age group. The current study and Chastin et al. [27] use the same cut-off points to identify sedentary time and intensities of activity. Therefore, the higher time spent sedentary in the current study is likely to be a true, and somewhat concerning difference. It is possible that the sedentary behavior was noticeably higher in our study because adolescents contribute less to the running of a household than adults and have homework to complete that requires them to be sedentary. While the total sedentary time of the study population is concerning, on a positive note, it does not seem to be accumulated in prolonged bouts, and is interspersed with bouts of light and moderate-vigorous activity. Therefore, it seems unlikely that these adolescent girls are exposing themselves to the additional harmful effects of prolonged sedentary time [28].

Individual components of the 24-h day (e.g., physical activity) have been assessed across different BMI categories in previous studies, however, there is limited evidence reporting on the entire 24-h activity patterns. The present study shows every 1 BMI z-score higher was associated with 16 min more in LPA, and 156 cpm lower MVPA intensity. The higher LPA is a somewhat surprising and unexpected result, however it is similar to results reported in other studies [27,29]. Chastin et al. [27] used Actigraph accelerometers on an adult sample to present the composition of a 24-h day for participants in different BMI categories. Compared to the overall compositional mean, in the obese group, the proportion of time spent in LPA was greater by 6% whereas MVPA was lower by 20%. There is a strong debate surrounding the benefits of LPA, some authors classify LPA as inactivity because it displaces time spent in MVPA [30], whereas others highlight the potentially beneficial effect of engaging in LPA rather than sedentary behavior [31,32]. Regardless of the direction of the association, it would seem that targeting sleep and MVPA in overweight and obese participants would lead to better health outcomes.

Prior research suggests components of the whole 24-h day matters [3], and synergistic effects of adhering to all of the 24-h movement guidelines provides higher benefits than meeting individual guidelines [3,26,33]. In the present study approximately one fifth (19.3%), one quarter (22.7%), and 7% of participants met the recommended sleep guidelines, MVPA guidelines and both, respectively. Additionally, healthy weight individuals were more likely to meet the sleep (23.1%) and MVPA (15.4%) guidelines compared to their overweight (16.1% and 9.7%) and obese (0% and 11.1%) counterparts, respectively. Poor adherence to the screen time recommendation of <2 h of recreational screen time per day is commonly reported but was not assessed in this study [34,35,36,37].

Recently, there has been some suggestion that, particularly in girls, physical education teacher support is more important than qualitative goals at enhancing physical activity [38]. Interestingly, in New Zealand physical education is not a compulsory subject after year 10, and only 36% of this sample reported being enrolled in physical education as a subject at school; although, 68% reported participating in organized sport (data not shown). Further investigation into how participation in physical education and organized sport effects both MVPA and other components of the 24-h day are warranted. 

This is one of only a small number of studies to measure 24-h activity patterns with accelerometry and the first to do so in NZ adolescents. Participants included a wide-spread sample of female adolescents from eight locations across NZ, providing a more heterogeneous sample than single-site studies. However, this study has a number of limitations: our sample size was limited by the design of the study to primarily assess nutrient intakes with the measurement of 24-h activity an optional extra for participants. Furthermore, our sample was not representative of all of NZ female adolescents. We chose to use the adult Freedson cut points (which are validated for accelerometer placement at the hip) as very few algorithms have been validated for the age group studied (the upper age range for many validations is 16 years) and we assumed that the physical size of our 16–18-year-old participants would be more similar to adults than younger adolescents. Differences in placement of accelerometers, epoch lengths, cut points and sleep algorithms, wear time and valid data identification limit comparisons between studies. There is a need for definitive consensus in this area to facilitate direct comparisons between populations. The accelerometers were unable to differentiate between different sedentary behaviors (e.g., screen-time vs. non-screen time); therefore, the prevalence of those meeting the screen-time recommendation is unable to be calculated. Hip-worn Actigraph accelerometers also lack the ability to distinguish posture. For example, a person standing still may be classified as sedentary, even though sedentary behavior is defined as being in a seated position [39]. However, accelerometry is considered less biased and more robust than self-reports and is clearly a superior tool for the measurement of 24-h activity. For simplicity, associations between 24-h components and BMI were not adjusted for changes in other components of the 24-h day (e.g., the association between BMI and MVPA was not adjusted for sleep, LPA or sedentary time). Future studies may consider investigating these associations using compositional data analysis. Our population sample was 15–18 year old females. The current guidelines recommend adolescents (12–17 years) attain at least 60 min/day of MVPA, compared to adult (18–65 years) guidelines of at least 150 min/week of MVPA. For study convenience and due to a small percentage of 18-year-olds (*n* = 5), we chose to compare the whole sample to the youth guidelines. However, four of these participants did at least 30 min of MVPA per day, therefore it would increase the prevalence of those meeting the physical activity guidelines from 22.7% to 24.4%.

## 5. Conclusions

To our knowledge, this is the first study to measure 24-h movement activity in NZ adolescent females. The present study highlights that adolescent females aged 15—18 years in NZ are spending approximately half their time sedentary. Additionally, a higher BMI z-score is associated with more LPA, and a reduced likelihood of meeting either the MVPA guidelines, the sleep guidelines, or both. Persisting with these inactive behavioral choices among adolescents could have detrimental effects on the health of this population with a potentially increased development of chronic disease. Understanding the prevalence of adhering to 24-h guidelines rather than individual guidelines will be crucial to the planning and development of public health policies and interventions.

## Figures and Tables

**Table 1:** Associations between body mass index (BMI) and different components of a 24-h day, measured in 119 New Zealand adolescent females.

	Mean Difference (95% CI) for each z-Score Higher BMI	Mean Difference (95% CI) for Overweight Compared to Healthy Weight	Mean Difference (95% CI) for Obese Compared to Healthy Weight
**24 h components**			
Sleep (minutes)	−11 (−23, 2)	−16 (−42, 10)	−28 (−72, 16)
Sedentary time (minutes)	−3 (−22, 16)	−7 (−47, 34)	4 (−64, 71)
LPA (minutes)	**−16 (3, 29)**	24 (−4, 52)	34 (−13, 81)
MVPA (minutes)	−4 (−8, 1)	−7 (−16, 2)	−11 (−27, 4)
**Breaks in sedentary time**			
Number of breaks	2 (−3, 6)	5 (−4, 13)	−2 (−16, 12)
Mean duration of breaks (minutes)	−0.3 (−1.0, 0.4) ^a^	−0.6 (−2.0, 0.9) ^a^	0.1 (−2.3, 2.6) ^a^
Mean intensity of breaks (counts per min)	5 (−29, 40)	−48 (−121, 26)	−2 (−124, 120)
Mean duration of uninterrupted sedentary time (minutes)	−6 (−23, 11)	−14 (−51, 23)	8 (−53, 70)
**Bouts of MVPA**			
Number of bouts	0 (−1, 1)	−1 (−4, 2)	−1 (−5, 4)
Mean duration of bouts (minutes)	−0.1 (−0.3, 0.05)	−0.2 (−0.6, 0.2)	−0.5 (−1.1, 0.2)
Mean intensity of bouts (counts per min)	**−156 (−301, −10)**	−188 (−494, 118)	**−517 (−1027, −6)**

^a^ Median difference (95% CI)—median regression used due to outlier. Bold means and 95% CIs are statistically significant.

**Table 2:** Numbers of participants meeting the sleep and physical activity guidelines by weight status.

	N (%) Meeting Sleep Recommendation (8–10 h a Night)	N (%) Meeting Physical Activity Recommendation (60+ min of MVPA a Day)	N (%) Meeting Both Sleep and Physical Activity Recommendations
Whole sample	23 (19.3)	27 (22.7)	8 (6.7)
Healthy weight (*n* = 78)	18 (23.1)	21 (26.9)	6 (7.7)
Overweight (*n* = 32)	5 (15.6)	5 (15.6)	2 (6.3)
Obese (*n* = 9)	0	1 (11.1)	0
